# Early detection of infectious complications using C-reactive protein and the procalcitonin levels after laparoscopic colorectal resection: a prospective cohort study

**DOI:** 10.1007/s00595-020-02111-6

**Published:** 2020-08-12

**Authors:** Teppei Tatsuoka, Takashi Okuyama, Emiko Takeshita, Haruka Oi, Takuji Noro, Takashi Mitsui, Hideyuki Yoshitomi, Masatoshi Oya

**Affiliations:** Department of Surgery, Saitama Medical Center, Dokkyo Medical University, 2-1-50 Minami-Koshigaya, Koshigaya, Saitama 343-8555 Japan

**Keywords:** Laparoscopic surgery, Colorectal cancer, Postoperative infection, C-reactive protein, Procalcitonin

## Abstract

**Purpose:**

The predictive values of the C-reactive protein (CRP) and procalcitonin (PCT) levels for postoperative infectious complications were investigated in patients who underwent elective laparoscopic resection of colorectal cancer.

**Methods:**

A total of 154 consecutive patients who underwent elective laparoscopic resection for colorectal cancer (CRC) were prospectively studied. The CRP and PCT levels on the first postoperative day (POD1) and the fourth postoperative day (POD4) were measured. Any correlations between the CRP and PCT levels on POD1 and POD4 with the occurrence of infectious complications were examined.

**Results:**

Infectious complications occurred in 18 (11.7%) patients. CRP on POD1 and CRP and PCT on POD4 were significantly higher in patients who developed infectious complications than in those who did not. The areas under the receiver operating characteristic curves of CRP on POD1 and CRP and PCT on POD4 were 0.597, 0.763 and 0.768, respectively. The cut-off values of CRP and PCT levels on POD4 were 14.33 mg/dl and 0,264 ng/ml, respectively. Whereas the positive predictive value of an elevated CRP level was high, the negative predictive value of an elevated PCT was high.

**Conclusion:**

The CRP and PCT levels on POD4 are both considered to be useful for the early detection of infectious complications after laparoscopic resection of CRC.

**Electronic supplementary material:**

The online version of this article (10.1007/s00595-020-02111-6) contains supplementary material, which is available to authorized users.

## Introduction

Infectious complications are relatively common after colorectal resection even in high-volume surgical centers [1, 2]. Intra-abdominal infections such as anastomotic leakage and intra-abdominal abscess in particular may cause sepsis and even result in postoperative death.

In recent years, laparoscopic surgery for colorectal resection has become very prevalent. It is usually combined with the early recovery after surgery protocol, and some of these patients can develop infectious complications after hospital discharge [3]. In such cases, a delay in the diagnosis and appropriate treatment for infectious complications may result in very serious outcomes.

The usefulness of inflammatory makers including C-reactive protein (CRP) and procalcitonin (PCT) has been described in several reports including a meta-analysis [4, 5]. However, analyses confined to laparoscopic surgery are limited [3]. In the present study, the predictive values of the CRP and PCT levels for postoperative infectious complications after laparoscopic colorectal resection performed at a single surgical department were explored.

## Patients and methods

The study included 154 consecutive patients (94 men, 50 women) who had undergone elective laparoscopic resection of colorectal cancer in the Department of Surgery at Saitama Medical Center, Dokkyo Medical University, between December 2018 and November 2019. Sixteen patients underwent robot-assisted surgery and the remaining 138 patients underwent conventional laparoscopic surgery. Patients who underwent conversion from laparoscopic to conventional open surgery, and those who underwent synchronous resection of hepatic metastasis were excluded from the present investigation. The median age of the patients was 70.7 years (range, 34–87 years). The surgical procedures performed for the patients are shown in Table 1.

**Table 1:** Performed surgical procedures in the present study

Performed surgical procedures	
Colon	
Ileocecal resection	25
Right hemicolectomy	25
Transverse colectomy	4
Left hemicolectomy	9
Sigmoid colectomy	36
Anterior resection	12
Total colectomy	1
Rectum	
Low anterior resection	20
Very low anterior resection	11
Abdominoperineal resection	9
Hartmann operation	2

To measure the serum CRP and PCT levels, peripheral blood was obtained from the cubital vein in the morning on postoperative day (POD1) and POD4. The serum CRP level was measured by latex immunoturbidimetry using a BM6070 (Japan Electron Optics Laboratory, Tokyo, Japan). The serum PCT level was determined by performing an electro-chemiluminescence immunoassay using a Cabas 8000 device (Rosch Diagnostic K.K, Tokyo, Japan) in the routine manner in the clinical laboratory of the hospital. The measurement of the PCT level on POD4 was missing in 18 patients because it was not included in the routine postoperative blood testing protocol. In the present study, white blood cell count (WBC) measured in the routine postoperative laboratory test was also included as a possible co-factor.

Infectious complications were recorded mainly based on clinical symptoms such as fever, pain and tenderness, radiological findings and urinalysis. Intra-abdominal infection was considered present when at least one of the following criteria was met: the presence of pus or enteric contents within the drains, presence of an abdominal or pelvic collection on postoperative computed tomography (CT), and leakage of contrast media through the anastomosis during an enema or evident anastomotic dehiscence at reoperation for postoperative peritonitis. Wound infection including perineal wound infection after abdominoperineal resection was recorded when the wound was painful or erythematous with pustular discharge and/or a positive culture, the requiring the opening of the wound and the administration of antibiotics. The diagnosis of pneumonia was based on the radiological findings. Urinary tract infection was diagnosed by a positive urine culture and urine sediment findings requiring antibiotic treatment. The diagnosis of enteritis was made when stool cultures were positive for microbial pathogens requiring antibiotic treatment. Peristomal abscess and ischemia of the stoma requiring reoperation were included as infectious complication.

This study was approved by the institutional review board of Saitama Medical Center, Dokkyo Medical University (No. 1582). Before operation, all patients gave their written informed consent to have their postoperative sequential measurement of CRP and PCT levels measured and to undergo an analysis of the associations between their levels and postoperative complications.

### Statistical analysis

Comparisons of the CRP and PCT levels and WBC by the presence or absence of infectious complications were carried out using the Mann–Whitney *U* test. Receiver operating characteristic curve (ROC) analyses for the correlation of the CRP and PCT levels and WBC with infectious complications were performed, and the respective areas under the curve (AUCs) were calculated to evaluate the predictive values of the CRP and PCT levels and WBC. The optimal cut-off values were obtained from the largest Youden index (Sensitivity + Specificity-1) for each parameter [6]. Multivariate analyses of the CRP and PCT levels and WBC for the prediction of infectious complications were carried out using a multiple logistic regression analysis, with the occurrence of infectious complication as dependent variable, and CRP and PCT levels and WBC on POD1 and POD4 as independent variables. All statistical analyses were performed with the Dr. SPSS software package (SPSS Japan, Tokyo, Japan). *P* < *0.0*5 was considered to indicate a significant difference or correlation.

## Results

Postoperative infectious complications occurred in 18 patients (11.7%), of which 16 had surgical site infections (Table 2). Of the 18 patients, intra-abdominal infections such as anastomotic leak, intraperitoneal abscess and rectovaginal fistula occurred in 12 patients. The infectious complications were diagnosed on POD3 in one patient, on POD 4 in 5 patients, between POD5 and POD7 in 9 patients, and on POD8 or later in 3 patients. In 3 patients who had infectious complications, reoperation was performed. One patient after ileocolic resection who died of intestinal bleeding with intestinal ischemia that occurred on POD9 was not included in the group with infectious complications.

**Table 2:** Postoperative infectious complications in the present study

Postoperative infectious complications	
None	136
Surgical site infection	
Anastomotic leak	10
Intra-abdominal abscess	1
Rectovaginal fistula	1
Drain site infection	1
Wound infection	1
Peristomal infection	2
Other infectious complications	
Urinary tract infection	1
Enteritis	1

Table 3 compares the CRP and PCT levels and WBC between patients with and without postoperative infectious complications. On POD1, the CRP levels were significantly higher in patients with postoperative infectious complications. On POD4, the CRT and PCT levels and WBC were significantly higher in patients with postoperative infectious complications than in those without (Supplement Table 2).

**Table 3:** The comparison the CRP and PCT levels and the WBC between the patients with and without postoperative infectious complications

	Infectious complication ( +)		Infectious complication (−)		
	*n*	Median (interquartile range)	*n*	Median (interquartile range)	*p*
POD1					
CRP	18	6.72 (4.57–9.66)	136	5.15 (3.34–7.17)	0.045
PCT	18	0.522 (0.321–1.05)	136	0.425 (0.178–0.873)	0.184
WBC	18	8.1 (7–11.1)	136	8.6 (7.2–10.6)	0.613
POD4					
CRP	18	13.89 (4.25–20.18)	136	3.47 (2.28–6.92)	< 0.001
PCT	14	0.515 (0.274–1.3)	122	0.201 (0.114–0.418)	0.001
WBC	18	8.1 (6.8–9.4)	136	6.3 (5.2–7.8)	0.010

**Fig. 1:**
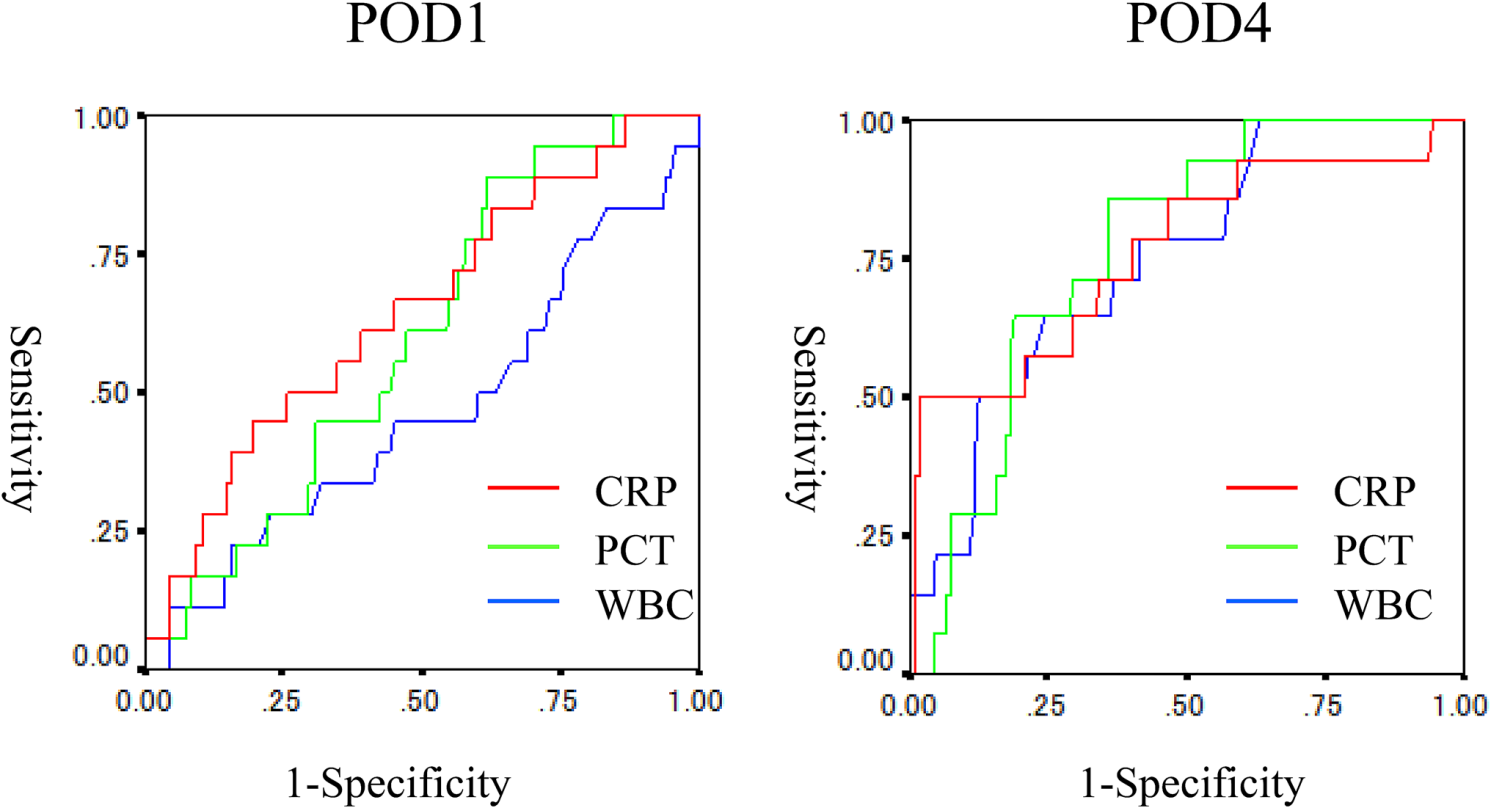
The ROC curves of CRP and PCT levels and WBC on POD1 and POD4

Figure 1 shows the ROC curves of CRP and PCT levels and WBC on POD1 and POD4. The AUCs of CRP and PCT levels and WBC on POD1 were 0.645, 0.597 and 0.463, with 95% confidence interval (CI) of 0.511–0.781, 0.476–0.717 and 0.311–0.615, respectively. The AUCs of CRP and PCT levels and WBC on POD4 were 0.763, 0.768 and 0.748, with a 95% CI of 0.614–0.913, and 0.666–0.870 and 0.625–0.871, respectively.

From the Youden index, the optimal cut-off value of CRT on POD1 was 9.95 mg/dl. The optimal cut-off values on POD 4 obtained were 14.33 mg/dl for CRP, 0.264 ng/ml for PCT and 6.750/μl for WBC. The sensitivity, specificity, positive predictive value (PPV) and negative predictive value (NPV) of CRP level on POD1, and CRP and PCT levels and WBC on POD4 calculated using these cut-off values are shown in Table 4. The PPV of CRP levels higher than 14.33 mg/dl on POD4 was 80.0%, whereas the NPV of PCT levels lower than 0.264 was 97.5%.

**Table 4:** The sensitivity, specificity, positive predictive value (PPV) and negative predictive value (NPV) of the CRP level on POD1, and the CRP and PCT levels and WBC on POD4 calculated using these cut-off values

	CRP on POD1 ≥ 7.95 mg/dl		WBC on POD4 ≥ 6750/μl		CRP on POD4 ≥ 14.33 mg/dl		PCT on POD4 ≥ 0.264 ng/ml	
Sensitivity	8/18	44.4%	14/18	77.8%	8/18	44.4%	12/14	85.7%
Specificity	110/136	80.9%	78/136	57.4%	134/136	98.5%	78/122	59.1%
PPV	8/34	23.5%	14/72	19.4%	8/10	80.0%	12/56	21.4%
NPV	110/120	91.7%	78/82	95.1%	134/144	93.1%	78/80	97.5%
Accuracy	118/154	76.6%	92/154	59.7%	142/154	92.2%	90/136	66.2%

Table 5 shows the results of multiple logistic regression analyses of the CRP and PCT levels and WBC on POD1 and POD4 for the occurrence of infectious complications. Whereas the CRP level on POD4 was a significant co-factor, the PCT level on POD4 was an independent and nearly significant co-factor. WBC on either POD1 or POD4 was not an independent co-factor (Supplement Table 1).

**Table 5:** The results of multiple logistic regression analyses of the CRP and PCT levels and WBC on POD1 and POD4 for the occurrence of infectious complications

	Odds ratio	95% Confidence interval		*p*
CRP ≥ 14.33 mg/dl vs CRP<14.33 mg/dl	29.40	4.74	182.29	< 0.001
PCT ≥ 0.264 ng/ml vs PCT<0.264 ng/ml	4.64	0.86	24.95	0.074

The relationships between combinations of the CRP and PCT levels on POD4 and postoperative infectious complication are shown in Table 6. None of the patients with CRP levels 14.33 mg/dl or higher had PCT levels higher than 0.264 ng/ml. Thus, patients with CRP levels lower than 14.33 mg/dl and PCT levels higher than 0.264 ng/ml had an intermediate risk of infectious complications.

**Table 6:** The relationships between combinations of the CRP and PCT levels on POD4 and postoperative infectious complication

	Infectious complication	
	Present	Absent
CRP ≥ 14.33 mg/dl and PCT ≥ 0.264 ng/ml	7	2
CRP ≥ 14.33 mg/dl and PCT<0.264 ng/ml	0	0
CRP<14.33 mg/dl and PCT ≥ 0.264 ng/ml	5	42
CRP<14.33 mg/dl and PCT<0.264 ng/ml	2	78

Table 7 explores the clinical factors which may influence the CRT and PCT levels on POD4. Elevation of CRP level on POD4 was marginally more frequent in male patients than in female patients. An elevation of the PCT level on POD4 was marginally more frequent in the elderly patients. However, using a multiple logistic regression analysis including age and gender as co-factors, the occurrence of postoperative infection was an independent co-factor associated with elevated CRP and PCT levels on POD4. Tumor site (colon/rectum), body mass index, duration of operation and intraoperative bleeding were not associated with the CRP and PCT levels on POD4.

**Table 7:** Clinical factors which may influence the CRT and PCT levels on POD4

	CRP on POD4			PCT on POD4		
	≥ 14.33 mg/dl	<14.33 mg/dl	*p*	≥ 0.264 ng/ml	<0.264 ng/ml	*p*
*n*	10	144		56	80	
Age^a^	70.6 (66.5–75.3)	70.7 (63.1–76.5)	0.846	71.7 (67.2–76.4)	70.1 (60.2–76.0)	0.067
Gender (male: female)	9:1	85:59	0.052	32:24	48:32	0.739
BMI^a^	22.1 (21.4–26.4)	23.3 (20.3–25.0)	0.739	22.8 (20.9–25.5)	23.2 (20.5–24.8)	0.654
Tumor site (colon: rectum)	5:5	107:37	0.095	41:15	57:23	0.802
Duration of operation (min)^a^	175.5 (147.0–230.3)	181 (156.3–261)	0.548	175.5 (147–23.3)	181 (156.3–261)	0.486
Intraoperative bleeding (g)^a^	10 (5–475)	12.5 (5–30)	0.662	10 (5–47.5)	12.5 (5–30)	0.662
Infection complications (present: absent)	8:2	10:134	< 0.001	12:44	2:78	< 0.001

^a^Continuous variables

## Discussion

Over the last decade, various inflammatory makers have been suggested to assist in the early diagnosis of postoperative infections. Among these, CRP and PCT have been the most commonly used, and, they overall have been identified as good predictors [3, 5, 7–13]. The results of the current study showed that both the CRP and PCT levels on POD4 predicted infectious complications after laparoscopic colorectal resection. According to multivariate analyses using a logistic regression analysis, the predictive values of CRP and PCT on POD4 were nearly independent. Although WBC on POD4 was significantly higher in patients with infectious complication than in those without infectious complication, it was not an independent predictor. Using cut-off values of 14.33 mg/dl for CRP, and 0.264 ng/ml for PCT on POD4, the PPV of an elevated CRP on POD4 was high (80%) whereas the NPV of a low PCT on POD4 was high (97.5%). These results suggest that a high CRP on POD4 is useful to identify patients who are likely to develop postoperative infectious complications and that a low PCT on POD4 suggests a postoperative course without infectious complications.

Several previous reports have so far compared the predictive values of CRP and PCT levels for postoperative complications in patients undergoing colorectal surgery with conflicting results. Some studies showed the superiority of the PCT level over the CRP level or the usefulness of the combined analysis of the CRP and PCT levels [8, 9]. In contrast, others reported the superiority of the CRP level over the PCT level or equal predictive values of the CRP and PCT levels [3, 7, 10, 11].

In the present study, the AUCs of the ROC curves of CRP on POD4 and of PCT on POD4 did not differ (0.763 versus 0.768). A meta-analysis by Cousin et al. [4] concluded that the routine measurement of PCT for the prediction of infectious complications had no additional value, because the cost of PCT measurement is much higher than that of CRP. In contrast, a recent systematic review by Tan et al. reported that PCT on POD5 could guide safe discharge after colorectal surgery [5]. Giaccaglia reported that the addition of PCT to CRP provided better ROC curves than PCT or CRP alone [11].

In the current study, the cut-off value of CRP on POD4 calculated using the Youden index was 14.33 mg/dl, slightly higher than the cut-off values in the previous studies [10, 12]. Although the sensitivity of 44.4% was quite low, the positive predictive value of 80% was high. The cut-off value of 0.264 ng/ml for PCT on POD4 was similar to that in a previous study [10] and showed a very high NPV. Actually, the shapes of the ROC curves of CRP and PCT on POD4 were slightly different, though the AUCs were similar. These results suggest that the combination of CRP and PCT level on POD4 is more useful than either the CRP or the PCT level alone.

Since most previous studies included patients who underwent both open surgery and laparoscopic surgery, analyses of patients after laparoscopic surgery have been few [3]. Facy et al. reported that a CRP cut-off value of 10 mg/dl on POD4 could be applied to both open and laparoscopic colorectal surgeries [12] because they did not find any significant differences in the CRP and PCT levels on POD4 between patients after open surgery and those after laparoscopic surgery if they had intra-abdominal infections. However, laparoscopic surgery is reportedly associated with better short-term clinical and inflammatory outcomes [14, 15]. Laparoscopic surgery has been used for the majority of elective colorectal resection in recent years. Indeed, more than 80% of elective resections of colorectal cancer were carried out laparoscopically in our department (data not shown). Therefore, exploring the cut-off values only for patients undergoing laparoscopic surgery is thought to be worthwhile (Supplement Table 3). Thus, only patients after laparoscopic surgery were included in the present study.

In the clinical setting, PPV and NPV are often more important than sensitivity and specificity in the decision making. All patients with CRP levels on POD4 over 14.33 mg/dl had PCT levels on POD4 over 0.264 ng/ml. The PPV of a CRP on POD4 level over 14.33 mg/dl was 80%, suggesting that patients having such high levels of CRP on POD4 are very likely to develop infectious complication on POD4 or later. In contrast, the NPV of a CRP level 14.33 mg/dl or higher on POD4 was 93.1%, suggesting that 6.9% of patients with CRP levels lower than 14.33 mg/dl on POD4 developed infectious complications. Since postoperative infectious complications occurred in 18 of 154 patients (11.7%), a false negative rate of 6.9% for CRP on POD4 level might be important. In contrast, only 2 of 80 (2.5%) patients with PCT levels lower than 0.264 ng/ml on POD4 developed infectious complication. This high NPV appears to be clinically useful because the majority of such patients can be safely discharged especially in the enhanced recovery after laparoscopic surgery protocol.

Patients with CRP levels higher than 14.33 mg/dl on POD4, even without clinical symptoms, are considered to have an increased risk of infections, and they should be carefully observed until infectious complications are completely ruled out. The removal of intra-abdominal drains should therefore be done very carefully. If they have clinical symptoms such as fever or pain, oral intake should be suspended, and computer tomography (CT) is recommended to detect any areas of abdominal fluid collection, excessive intra-abdominal free air, and pneumonia to start early treatment.

In our department, postoperative meal intake was started on POD3 according to the routine postoperative recovery protocol. In the patients of the present study, postoperative oral meal intake was already started at the morning of POD4 in 8 out of 12 patients who developed intra-abdominal infections (anastomotic leak, abscess or rectovaginal fistula). The oral meal intake was stopped on POD4 in 3 patients, and later in 4 patients. The CRP and PCT levels on POD4 were practically utilized in the 3 patients for the determination of suspension of oral intake on POD4 in combination with the clinical symptoms.

The timing of the measurement of the CRP or PCT levels for prediction should be discussed. Although some previous studies measured the CRP or PCT levels daily after operation [3, 7, 8, 12, 16], daily measurement is not practical because of its cost. The largest AUC of the ROC curves were obtained for both CRP and PCT levels between POD3 and POD5 in the previous studies [4, 7, 8, 10, 11]. In the present study, CRP and PCT levels were measured on POD1 and POD4, when routine blood tests were scheduled. Since elective colorectal surgery is performed on Monday, Thursday and Friday in our hospital, POD1 and POD4 are usually regular working days. In addition, infectious complications were clinically diagnosed on POD3 in one patient, on POD4 in 5 patients and between POD5 and POD7 in 9 patients, suggesting that the CRP and PCT levels on POD7 are not predictive in the majority of patients with infectious complications.

One limitation associated with the current study is that PCT on POD4 was not measured in 18 patients including 4 patients who developed infectious complications. Although CRP on POD4 was measured as a routine postoperative blood test in 154 consecutive patients, measurement of PCT was not included as a routine blood test on POD4. The missing data for PCT on POD4 in 18 patients might have biased the results. In addition, the results of the present study should be validated using a separate group of patients. For the validation and also for the application as a clinical practice, the measurement of CRP and PCT levels on POD4, and the investigation on the relationship with postoperative infectious complication are continued in patients operated later than December 2019 in our department.

In conclusion, the current study suggests that the measurement of both the CRP and PCT levels on POD4 is useful for predicting postoperative infectious complications after laparoscopic colorectal resection, and that the combination of the CRP and PCT levels on POD4 may enhance the utility of these markers in the postoperative management of patients after laparoscopic colorectal resection.

## Electronic supplementary material

Below is the link to the electronic supplementary material.

Supplementary file1 (DOCX 26 kb)

## Data Availability

The datasets supporting the conclusion of this article are included within the article. The underlying datasets are available from the corresponding author on reasonable request.
