# Supplementary material for: Early detection of infectious complications using C-reactive protein and the procalcitonin levels after laparoscopic colorectal resection: a prospective cohort study
Source: Surg Today. 2020 Aug 12;51(3):397–403. doi: 10.1007/s00595-020-02111-6 (PMC7892676; doi:10.1007/s00595-020-02111-6)
Supplement: Supplementary file 1 — Supplementary file1 (DOCX 26 kb) [file 595_2020_2111_MOESM1_ESM.docx]

Supplement Table1

The predictive value of CRP and PCT levels and WBC on POD1 and POD4 by the multiple logistic regression analysis for the occurrence of infectious complications

Supplement Table2

The differences of CRP and PCT levels and WBC on POD1 and POD4 between intra-abdominal infections and other inflammatory complications

Supplement Table3

The complications of CRP and PCT levels and WBC on POD1 and POD4 between patients after laparoscopic surgery and open surgery, including both with and without infection complications
